# Tomato SlRbohB, a member of the NADPH oxidase family, is required for disease resistance against *Botrytis cinerea* and tolerance to drought stress

**DOI:** 10.3389/fpls.2015.00463

**Published:** 2015-06-23

**Authors:** Xiaohui Li, Huijuan Zhang, Limei Tian, Lei Huang, Shixia Liu, Dayong Li, Fengming Song

**Affiliations:** National Key Laboratory for Rice Biology, Institute of Biotechnology, Zhejiang University, HangzhouChina

**Keywords:** tomato (*Solanum lycopersicum* L.), respiratory burst oxidase homologs (Rbohs), *Botrytis cinerea*, defense response, drought stress tolerance

## Abstract

NADPH oxidases (also known as respiratory burst oxidase homologs, Rbohs) are key enzymes that catalyze the generation of reactive oxygen species (ROS) in plants. In the present study, eight *SlRboh* genes were identified in tomato and their possible involvement in resistance to *Botrytis cinerea* and drought tolerance was examined. Expression of *SlRbohs* was induced by *B. cinerea* and *Pseudomonas syringae* pv. *tomato* but displayed distinct patterns. Virus-induced gene silencing based silencing of *SlRbohB* resulted in reduced resistance to *B. cinerea* but silencing of other *SlRbohs* did not affect the resistance. Compared to non-silenced plants, the *SlRbohB*-silenced plants accumulated more ROS and displayed attenuated expression of defense genes after infection with *B. cinerea*. Silencing of *SlRbohB* also suppressed flg22-induced ROS burst and the expression of *SlLrr22*, a marker gene related to PAMP-triggered immunity (PTI). Transient expression of *SlRbohB* in *Nicotiana benthamiana* led to enhanced resistance to *B. cinerea*. Furthermore, silencing of *SlRbohB* resulted in decreased drought tolerance, accelerated water loss in leaves and the altered expression of drought-responsive genes. Our data demonstrate that SlRbohB positively regulates the resistance to *B. cinerea*, flg22-induced PTI, and drought tolerance in tomato.

## Introduction

Reactive oxygen species (ROS) are known to participate in various cellular mechanisms and play multiple signaling roles in a wide range of organisms (Suzuki et al., 2011; Marino et al., 2012; Baxter et al., 2014; Kaur et al., 2014). Growing evidence indicates that ROS such as superoxide anion and hydrogen peroxide (H_2_O_2_) are important signaling molecules that regulate a broad range of biological processes involved in growth, development, and responses to abiotic and biotic stresses (Torres et al., 2006; Torres, 2010; Mittler et al., 2011; Marino et al., 2012; Nathan and Cunningham-Bussel, 2013; Baxter et al., 2014; Kaur et al., 2014).

In plants, ROS are mainly generated by a number of enzymes (Apel and Hirt, 2004; Tripathy and Oelmuller, 2012). NADPH oxidases, also known as respiratory burst oxidase homologs (Rbohs), are the most extensively studied ROS-generating enzymes (Sagi and Fluhr, 2006; Suzuki et al., 2011). Rbohs are localized on the plasma membrane; however, dynamic changes in the subcellular localization of tobacco NtRbohD in response to elicitor treatment were recently reported (Noirot et al., 2014). Generally, Rbohs form enzymatic complexes and catalyze the production of superoxide radicals via FAD and two independent hemes (Sagi and Fluhr, 2006). It was suggested that plant and animal Rbohs share a common mechanism for activation of their enzymatic activity upon developmental and stress signals (Canton and Grinstein, 2014). The activity of plant Rbohs was found to be regulated in different ways, e.g., the binding of Rac GTPase to the N-terminal extension (Wong et al., 2007; Oda et al., 2010), protein modification via phosphorylation (Xing et al., 2001; Kobayashi et al., 2007; Ogasawara et al., 2008; Sirichandra et al., 2009; Yoshioka et al., 2009; Kimura et al., 2012; Takahashi et al., 2012; Dubiella et al., 2013), *S*-nitrosylation (Yun et al., 2011), and extracellular ATP and phospholipid signaling (Song et al., 2006; Demidchik et al., 2009; Zhang et al., 2009).

Plant Rbohs constitute a multigene family and have been identified in a wide range of plant species (Kaur et al., 2014). The Rboh family is comprised of 10 members in *Arabidopsis*, named AtRbohA-H, and members in rice (Sagi and Fluhr, 2006; Wong et al., 2007). Recent studies have revealed that plant Rbohs are involved in a multitude of different signaling pathways that regulate root hair growth, stomatal closure, pollen–stigma interactions, defense responses to pathogens, and acclimation to abiotic stresses (Torres, 2010; Suzuki et al., 2011; Marino et al., 2012; Nathan and Cunningham-Bussel, 2013; Baxter et al., 2014; Kaur et al., 2014). In *Arabidopsis*, AtRbohE was found to regulate the proper timing of tapetal-programmed cell death, a process that is critical for pollen development (Xie et al., 2014), and AtRbohH and AtRbohJ have been shown to modulate the amplitude and frequency of pollen tube growth and seed development (Müller et al., 2009; Kaya et al., 2014; Lassig et al., 2014). It was also found that the *Arabidopsis* AtRbohD, AtRbohF, and AtRbohC, maize Roothairless5 and cress LesaRbohB play roles in regulating lateral root development (Foreman et al., 2003; Jones et al., 2007; Macpherson et al., 2008; Muller et al., 2012; Jiao et al., 2013; Nestler et al., 2014; Li et al., 2015). The bean PvRbohB and *Medicago truncatula* MtRbohA were found to positively regulate nitrogen fixation and delay nodule senescence but negatively regulate AM colonization (Marino et al., 2011; Montiel et al., 2012; Arthikala et al., 2013, 2014). In addition to the functions in growth and development, Rbohs have been shown to play important roles in plant abiotic and biotic stress responses. The requirements for Rbohs in defense responses against different pathogens seem to be diverse in plant–pathogen interactions (Pogány et al., 2009; Torres et al., 2013). In *Arabidopsis*, AtRbohD and AtRbohF are required for the accumulation of ROS and function as critical regulators of defense responses (Torres et al., 2002, 2013; Maruta et al., 2011; Chaouch et al., 2012; Foley et al., 2013; Nozaki et al., 2013; Pastor et al., 2013). Although silencing of *AtRbohB* eliminated elicitin- and MAPK-mediated ROS generation (Asai et al., 2008), AtRbohD-regulated ROS burst is not linked to MPK3/MPK6 activation during early signaling events in plant immunity (Xu et al., 2014). In barley, silencing of *HvRbohA* or knockdown of *HvRbohF2* affected penetration by *Blumeria graminis* f. sp. *hordei* (Trujillo et al., 2006; Proels et al., 2010). The tobacco *NtRbohD* is responsible for ROS production in cryptogein-elicited cells and in herbivore-elicited responses (Simon-Plas et al., 2002; Lherminier et al., 2009; Wu et al., 2013), whereas silencing of *NbRbohA* and *NbRbohB* in *Nicotiana benthamiana* resulted in reduced ROS production in response to *Phytophthora infestans* (Yoshioka et al., 2003). Furthermoe, AtRbohD and AtRbohF were found to participate in ABA and ethylene signaling resulting in stomatal closure (Kwak et al., 2003; Bright et al., 2006; Desikan et al., 2006) and both of them have been shown to mediate rapid signaling that regulates abiotic stress responses (Miller et al., 2009; Xie et al., 2011; Ma et al., 2012).

Two Rboh genes, *SlRboh1* and *SlWfi1*, have been identified in tomato. These genes have been shown to be involved in wounding responses and development (Sagi et al., 2004). Recent studies revealed that SlRboh1 is required for brassinosteroid-induced apoplastic H_2_O_2_ production and stomatal closure/opening. Therefore, SlRboh1 plays an important role in acclimation-induced stress cross-tolerance (Zhou et al., 2012, 2014; Xia et al., 2014). However, the SlRboh family and the function of SlRbohs in biotic and abiotic stress responses are largely unknown. In the present study, we characterized the SlRboh family in tomato and investigated the possible involvement of *SlRbohs* in disease resistance and drought stress tolerance using a virus-induced gene silencing (VIGS) approach. Our VIGS-based functional analyses demonstrate that SlRbohB positively regulates the defense response against *Botrytis cinerea*, the flg22-induced immune response and tolerance to drought stress.

## Materials and Methods

### Plant Growth, Treatments, and Pathogen Inoculation

Tomato (*Solanum lycopersicum*) cv. Suhong 2003 was used for all experiments. Seedlings were grown in a mixture of perlite: vermiculite: plant ash (1:6:2) in a growth room under fluorescent light (200 μE m^2^s^-1^) at 22–24°C with 60% relative humidity and a 14 h light/10 h dark cycle. Pathogen inoculation, disease assays with *B. cinerea* or *Pseudomonas syringae* pv. *tomato* (*Pst*) DC3000 and the measurement of *in planta* fungal growth were basically performed according to previously described protocols (AbuQamar et al., 2008; Li et al., 2014a). Drought stress was applied to the plants by withholding watering for 2 weeks and stress phenotypes were recorded and photographed. For the measurement of water loss, fully expanded leaves were detached and water loss was measured according to a previously described method (Liu et al., 2014). Leaf samples were collected at the indicated time points after treatment or inoculation and used immediately for physiological and biochemical analyses or stored at −80°C until use.

### Identification of *SlRboh* Genes and Bioinformatics Analysis

*Arabidopsis* AtRbohs were used as queries to search against the tomato genomic database at the SOL Genomics Network (SGN) ^1^. The obtained candidate SlRboh sequences were examined using the domain analysis programs PFAM ^2^ and SMART ^3^ with the default cutoff parameters. The *Arabidopsis*, rice, and tomato Rboh protein sequences were aligned using the multiple alignment program MUSCLE 3.8.31 (Edgar, 2004). Maximum likelihood (ML) analyses were carried out using RAxML-HPC v.8 (Stamatakis, 2006; Stamatakis et al., 2008) on the XSEDE Teragrid of the CIPRES science Gateway (Miller et al., 2010) with default settings and JTT, followed by 1000 bootstrap replicates.

### Construction of VIGS and Transient Expression Constructs

For the VIGS constructs, fragments of 300–400 bp in sizes for *SlRbohs* were amplified from tomato cDNA using gene-specific primers (Supplementary Table S1), sequenced and then cloned into the TRV2 vector (Liu et al., 2002), yielding TRV2-*SlRbohA-H*. For the transient expression constructs, the *SlRbohB* coding sequence was amplified using the primers SlRbohB-GFP-F and SlRbohB-GFP-R (Supplementary Table S1) and cloned into pFGC-Egfp at *Xba*I/*Sma*I sites, yielding pFGC-*SlRbohB*. The recombinant plasmids TRV2-*SlRbohA-H* and pFGC-*SlRbohB* were introduced into *Agrobacterium tumefaciens* strain GV3101 by electroporation using the GENE PULSER II Electroporation System (Bio-Rad Laboratories, Hercules, CA, USA). Agrobacteria carrying different plasmids (TRV2-*SlRbohA-H* or pFGC-*SlRbohB*) were grown in YEP medium (50 μg ml^-1^ rifampicin, 50 μg ml^-1^ kanamycin, and 25 μg ml^-1^ gentamicin) for 24 h with continuous shaking at 28°C. Cells were collected by centrifugation and resuspended in infiltration buffer (10 mM MgCl_2_, 10 mM MES, and 200 μM acetosyringone at pH5.7).

### Agroinfiltration for VIGS and Transient Expression

For the VIGS assays, agrobacteria harboring TRV2-*SlRbohA-H* were mixed with agrobacteria carrying TRV1 at a ratio of 1:1 and adjusted to OD_600_ = 1.5. The mixed agrobacteria suspensions were infiltrated separately into the abaxial surface of 2-week-old seedlings using 1 ml needleless syringes (Liu et al., 2002). The VIGS-infiltrated plants were allowed to grow for 3 weeks before use in all experiments. For transient expression in *N. benthamiana*, agrobacteria carrying pFGC-*SlRbohB* or pFGC-eGFP empty vector were infiltrated into leaves of 4-week-old plants using 1 ml needleless syringes. Leaf samples were collected for analyzing the expression level of *SlRboh*B and for disease phenotyping and physiological, biochemical, and molecular analyses.

### qRT-PCR Analysis of Gene Expression

Total RNA was extracted using Trizol regent (TAKARA, Dalian, China) and treated with RNase-free DNase according to the manufacturer’s instructions. First strand cDNA was synthesized by reverse transcription using PrimeScript RT regent kit (TAKARA, Dalian, China) and the obtained cDNAs were used for gene expression analysis with qRT-PCR. Each qPCR reaction contained 12.5 μL SYBR Premix Ex TaqTM (TAKARA, Dalian, China), 0.1 μg cDNA and 7.5 pmol of each gene-specific primer (Supplementary Table S1) in a final volume of 25 μL, and performed on a CFX96 real-time PCR system (Bio-Rad, Hercules, CA, USA). Relative gene expression levels were calculated using 2^-ΔΔCT^ method (Livak and Schmittgen, 2001). Three independent biological replicates were analyzed.

### Western Blot Analysis

Extraction of total proteins from leaf samples, separation on SDS-PAGE gels and wet electroblotting transferring onto nitrocellulose membranes were carried out according to previously described protocols (Li et al., 2014a). GFP was detected using a mouse monoclonal GFP antibody (1:1000 dilution; No. M1210-1, Huaan Company, Hangzhou, China) and a peroxidase-conjugated anti-mouse antibody (1:8000 dilution; No. HA1008, Huaan Company, Hangzhou, China) according to the manufacturer’s instructions. Proteins on PVDF membranes were detected by SuperSignal West Pico Chemiluminescent Substrate (Pierce, Rockford, IL, USA).

### *In Situ* Staining and Measurement of H_2_O_2_

*In situ* staining of H_2_O_2_ was performed by 3, 3-diaminobenzidine (DAB) staining (Thordal-Christensen et al., 1997; Li et al., 2014a). The accumulation of H_2_O_2_ in stained leaves was visualized using a digital camera. Measurement of ROS burst in leaves was performed using a luminol-based luminescence method (Chakravarthy et al., 2010). Briefly, 4-mm leaf disks were floated in 200 μL ddH_2_O over night at room temperature in wells of a 96-well plate. Disks were then placed in 100 μL 400 nM flg22 solution containing 34 μg/ml of luminol (Sigma, St. Louis, MO, USA) and 20 μg of horseradish peroxidase (VI-A, Sigma, St. Louis, MO, USA) or in solution as a control. Luminescence was measured continuously at 1 min intervals for 30 min using a Synergy HT plate reader (BioTek Instruments, Inc. Winooski, VT, USA). Three replications were performed for each treatment.

### Statistical Analysis

All experiments were repeated independently three times. Data obtained from three independent experiments were subjected to statistical analysis according to Student’s *t*-test. Probability values of *p* < 0.05 were considered to represent significant differences.

## Results

### Identification of the *SlRbohs* Family in Tomato

**FIGURE 1:**
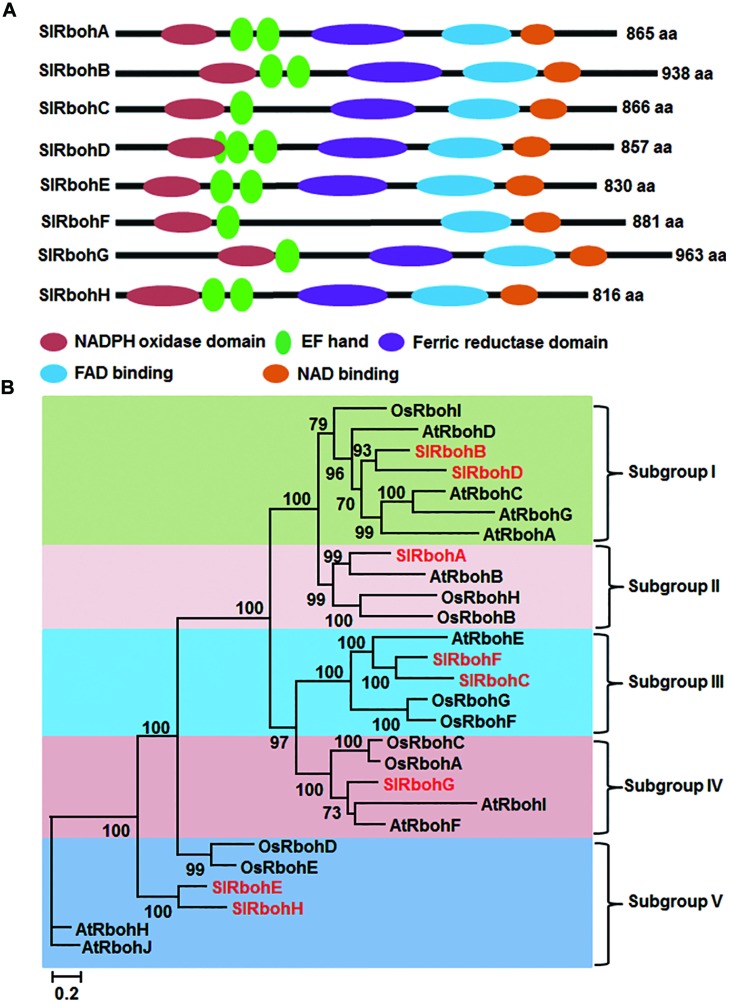
**Structure and phylogenetic tree of tomato Rboh proteins. (A)** Functional domains and their organization in SlRboh proteins. **(B)** Maximum-likelihood (ML) tree of SlRbohs with *Arabidopsis* AtRbohs and rice OsRbohs. The SlRboh proteins are indicated with red color. Only bootstrap values >50% are shown, and branch lengths are proportional to the number of substitutions per site (see scale bars).

Blastp searches against the tomato gnomic database using *Arabidopsis* AtRboh proteins as reference queries obtained nine significant hits corresponding to non-redundant putative *SlRboh* genes (**Table 1**). The predicted loci Solyc05g025680 and Solyc05g025690 are indeed the same gene, which encodes for SlRbohC. The SlRboh family therefore contains eight members, named SlRbohA-H (**Table 1**). *SlRbohB* and *SlRbohG* were previously reported as *SlWfi1* and *SlRboh1*, respectively (Sagi et al., 2004). By searching the SOL UniGene and NCBI databases, six out of eight *SlRboh* genes have full-length cDNA supports (**Table 1**). The sizes of the SlRboh proteins range from 816 (SlRbohH) to 963 (SlRbohG) amino acids with molecular weights of 94–109 kD and pIs of 8.63–9.09 (**Table 1**). Although the SlRboh proteins vary in size, they all share major functional domains with similar organization (**Figure 1A**); however, SlRbohF lacks the ferric reductase domain. The SlRboh proteins contain a conserved NADPH oxidase domain, 1–3 EF-hand domains, a ferric reductase domain, an FAD binding domain, and a NAD binding domain (**Figure 1A**). Phylogenetic analyses with *Arabidopsis* AtRbohs and rice OsRbohs indicate that SlRbohs can be classified into five subgroups (I–V; **Figure 1B**). Notably, each subgroup contains Rbohs from tomato, *Arabidopsis*, and rice. In the phylogenetic tree, SlRbohB and SlRbohD, with AtRbohA/C/D/G and OsRbohI, are assigned to subgroup I. SlRbohA, together with AtRbohB, OsRbohB/H, is classified into subgroup II. SlRbohC and SlRbohF are members of subgroup III, which contains AtRbohE and OsRbohF/G. SlRbohG belongs to subgroup VI, which includes AtRbohI/F and OsRbohA/C. The remaining proteins SlRbohE and SlRbohH, together with AtRbohJ/H and OsRbohE/D, are classified into subgroup V.

**Table 1:** Characterization of tomato NADPH oxidase genes.

Genes	Other names	Locus ID in SOL	Proteins in NCBI	cDNAs in SOL and NCBI	Size (aa)	MW (kD)	*p*I
*SlRbohA*		Solyc01g099620	XM_004230184	SGN-U586275, SGN-U601117	865	98.73	8.79
*SlRbohB*	SlWfi1	Solyc03g117980	NM_001247342	SGN-U579691, AF148534	938	105.31	9.08
*SlRbohC*		Solyc05g025690+ Solyc05g025680^a^	XM_004239534	NA	866	98.71	8.81
*SlRbohD*		Solyc06g068680	XM_004241593	SGN-U567947, SGN-U577640	857	97.73	9.01
*SlRbohE*		Solyc06g075570	XM_004242033	NA	830	95.40	9.02
*SlRbohF*		Solyc07g042460	XM_006353710	SGN-U573231, SGN-U575883	881	100.06	9.03
*SlRbohG*	SlRboh1	Solyc08g081690	NM_001288375	SGN-U564615, AF088276	963	109.08	9.09
*SlRbohH*		Solyc11g072800	XM_004251404	SGN-U572889	816	93.67	8.87

*^a^The predicted loci Solyc05g025690 and Solyc05g025680 are a single gene coding for SlRbohC.*

### Expression of *SlRbohs* in Response to *B. cinerea* and *Pst* DC3000

**FIGURE 2:**
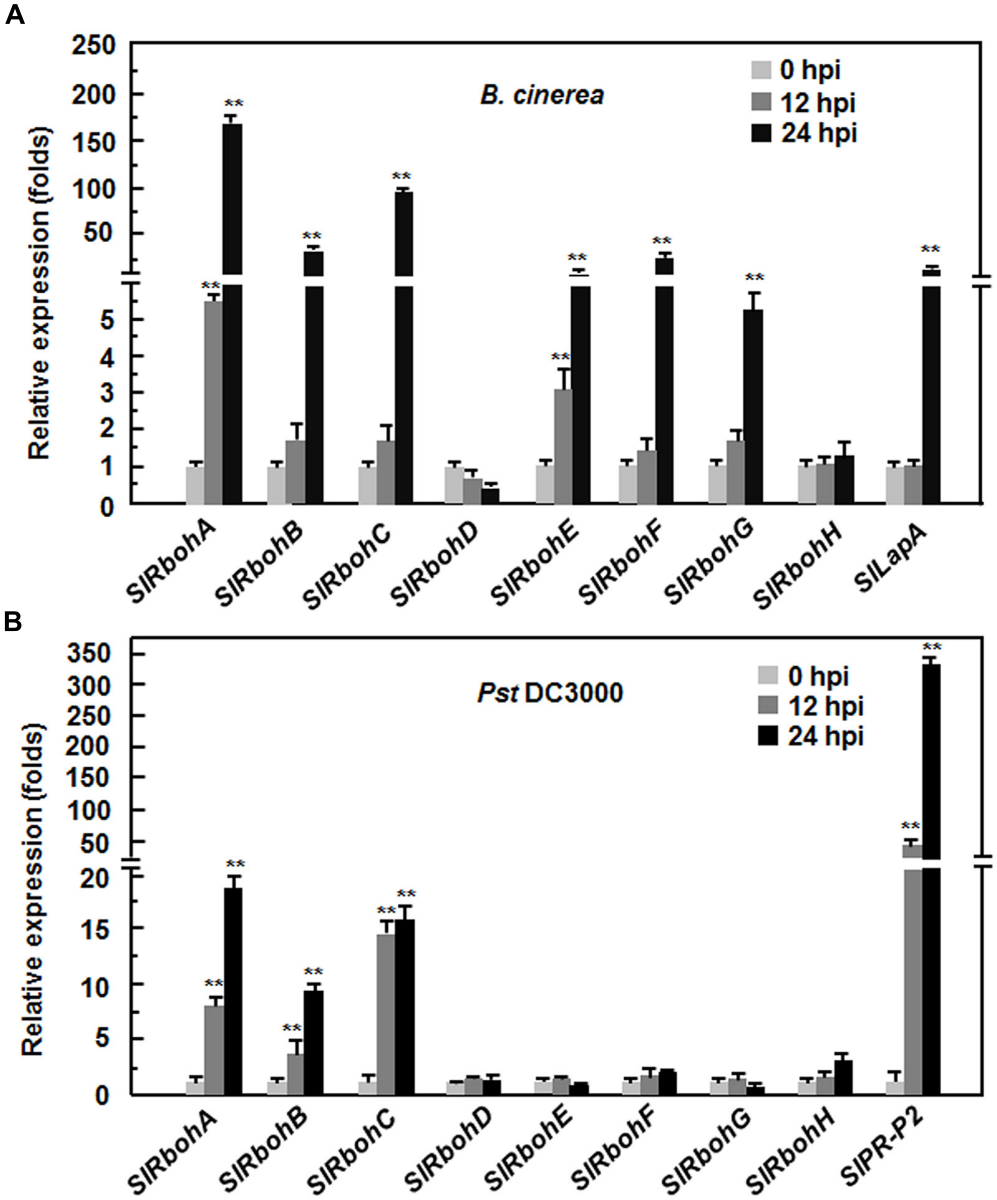
**Expression patterns of *SlRbohs* in response to *Botrytis cinerea* and *Pst* DC3000**. Tomato plants were inoculated by foliar spraying with spore suspension (2 × 10^5^ spores/mL) of *B. cinerea* **(A)** or vacuum infiltration with *Pseudomonas syringae* pv. *tomato* DC3000 (OD_600_ = 0.0002), **(B)** and leaf samples were collected at indicated time points. Gene expression was analyzed by qRT-PCR and relative expression levels were calculated by comparing with the corresponding values at 0 h (as a control) after inoculation. Data presented are the means ± SD from three independent experiments and ** above the columns indicate significant differences at *p* < 0.05 level.

To explore the possible involvement of *SlRbohs* in the defense response against pathogen, we analyzed and compared the expression patterns of *SlRbohs* in tomato plants after infection with *B. cinerea*, a necrotrophic fungal pathogen causing gray mold disease, or *Pst* DC3000, a (hemi)biotrophic bacterial pathogen causing bacterial leaf spot disease. To confirm the efficiency of the inoculation procedure, the expression patterns of *SlLapA*, a defense gene regulated by the JA/ET-mediated signaling pathway that is involved in the defense response against *B. cinerea*, and *SlPR-P2*, a defense gene regulated by the SA signaling pathway that is involved in the defense response against *Pst* DC3000, were monitored. As shown in **Figure 2**, the expression level of *SlLapA* in *B. cinerea*-inoculated plants showed a >40-fold increase at 24 hours post inoculation (hpi) while the expression level of *SlPR-P2* in *Pst* DC3000-inoculated plants displayed ~50-fold and >300-fold increases at 12 and 24 hpi, respectively. These findings indicate that the effectiveness of the inoculation procedure was satisfactory for further analysis of the expression patterns of *SlRbohs* in response to *B. cinerea* and *Pst* DC3000. After infection with *B. cinerea*, the expression of *SlRbohA*, *SlRbohB*, *SlRbohC*, *SlRbohE*, *SlRbohF*, and *SlRbohG* was significantly induced; the expression of *SlRbohD* and *SlRbohH* was not affected (**Figure 2A**). Among the *B. cinerea*-inducible *SlRbohs*, the expression levels of *SlRbohA* and *SlRbohE* started to increase at 12 hpi and peaked at 48 hpi, whereas the expression levels of *SlRbohB*, *SlRbohC*, *SlRbohF*, and *SlRbohG* only increased significantly at 24 hpi after infection with *B. cinerea*, as compared with those in the mock-inoculated plants (**Figure 2A**). After infection with *Pst* DC3000, the expression of *SlRbohD*, *SlRbohE*, *SlRbohF*, *SlRbohG*, and *SlRbohH* was not affected; the expression levels of *SlRbohA*, *SlRbohB*, and *SlRbohC* were significantly increased at 12 and 24 hpi compared to mock-inoculated plants (**Figure 2B**). These results indicate that *SlRboh* genes respond differentially to *B. cinerea* and *Pst* DC3000, showing different dynamics and magnitude of expression after pathogen infection.

### Silencing of *SlRbohB* Resulted in Reduced Resistance to *B. cinerea*

**FIGURE 3:**
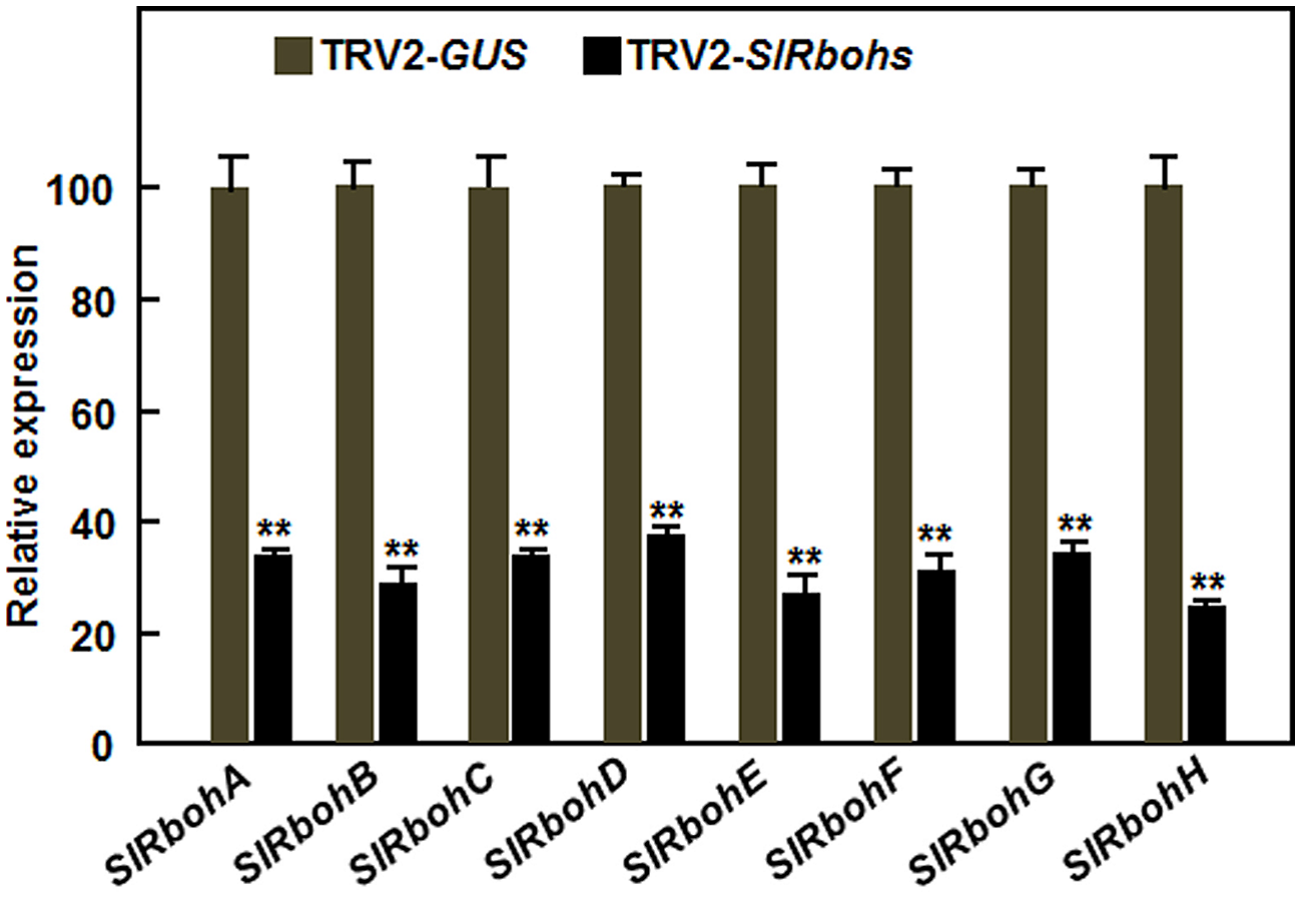
**Silencing efficiency of the *SlRboh* genes in silenced plants.** Two-week-old plants were infiltrated with agrobacteria harboring TRV2-*SlRbohA-H* or TRV2-*GUS* and leaf samples were collected at 3 weeks after VIGS infiltration. The silencing efficiency was calculated by comparing the transcript levels of the *SlRboh* genes in TRV2-*SlRbohs*-infiltrated plants with those in TRV2-*GUS*-infiltrated plants, which were set as 1. Data presented are the means ± SD from three independent experiments and ** above the columns indicate significant differences at *p* < 0.05 level.

Considering that relatively little is known about the function of Rbohs in resistance to necrotrophic fungal pathogens, we focused our efforts on exploring the possible involvement of *SlRbohs* in resistance to *B. cinerea* through VIGS-based functional analyses. For this purpose, standard VIGS procedure with a TRV2-*PDS* construct as an indicative for VIGS efficiency of each experiment was performed on 2-week-old tomato plants (Liu et al., 2002; Li et al., 2014a), followed by disease assays with *B. cinerea* at 4 weeks after VIGS infiltration. Under our experimental conditions, ~90% of TRV2-*PDS*-infiltrated plants showed a bleaching phenotype. Silencing efficiency for each *SlRboh* gene was evaluated by qRT-PCR and the transcript levels of the target *SlRboh* genes in the TRV2-*SlRboh*-infiltrated plants were compared to that in the TRV2-*GUS*-infiltrated plants. As shown in **Figure 3**, the transcript levels of the target *SlRboh* genes in the corresponding TRV2-*SlRboh*-infiltrated plants were significantly reduced and the silencing efficiency for the *SlRboh* genes was estimated to be 65–70%, indicating that the silencing efficiency with the designed constructs for each *SlRboh* gene under our VIGS procedure was appropriate for further experiments.

**FIGURE 4:**
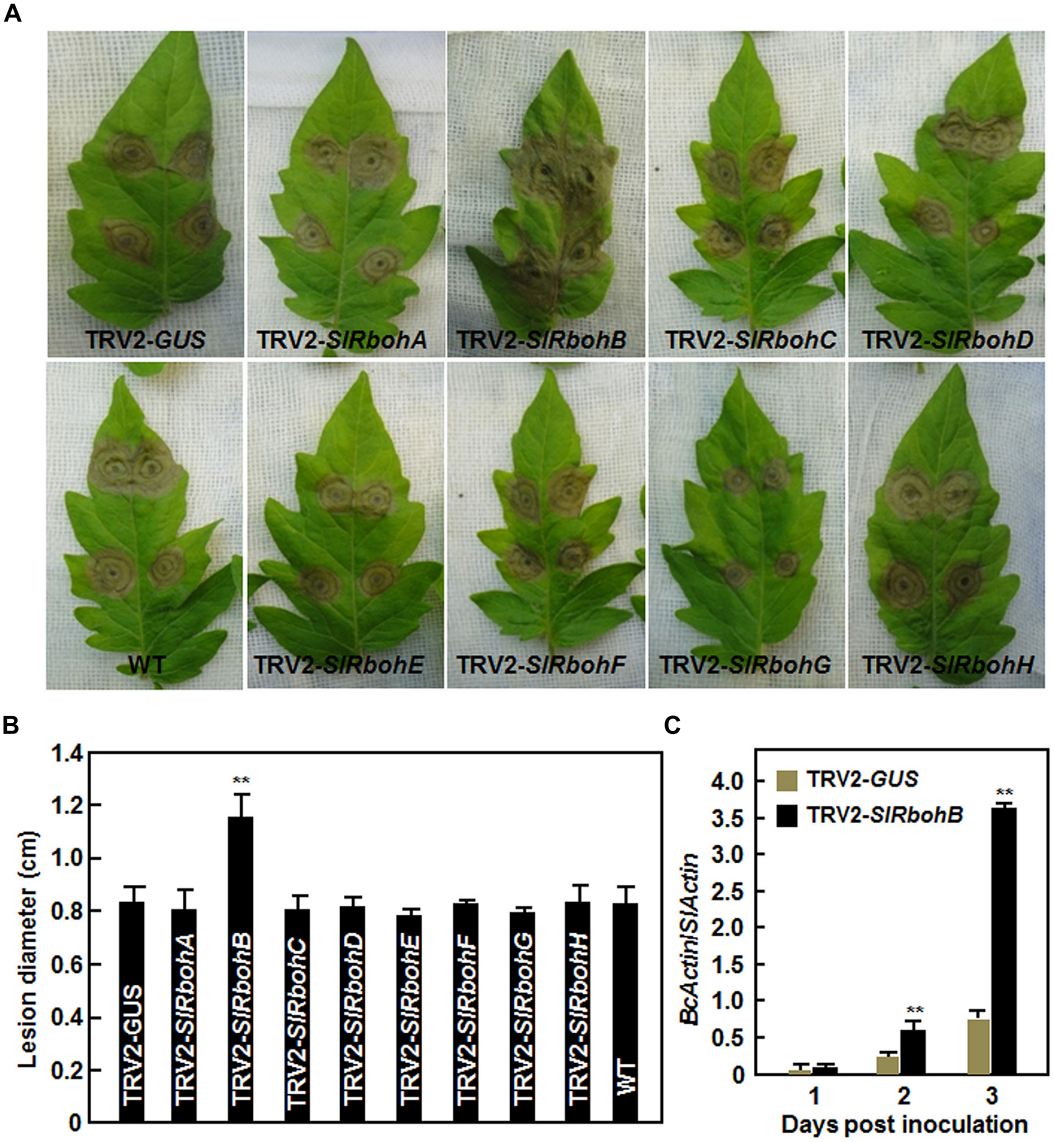
**Silencing of *SlRbohB* led to reduced resistance to *B. cinerea*. (A)** Disease symptom and **(B)** lesion size. Two-week-old plants were infiltrated with agrobacteria harboring TRV2-*SlRbohA–H* or TRV2-*GUS* and leaves were detached for disease assays at 4 weeks after VIGS infiltration. Inoculation was done by dropping spore suspension (1 × 10^5^ spores/mL) on detached leaves and lesion sizes were measured at 4 days after inoculation. A minimum of 20 leaves were included in each experiment. **(C)** *In planta* growth of *B. cinerea* in inoculated leaves. Fungal growth was assumed by analyzing the transcript level of *BcActin* gene by qRT-PCR using *SlActin* gene as an internal control and shown as the ratios of *BcActin*/*SlActin*. Data presented in **(B,C)** are the mean ± SD from three independent experiments and ** above the columns indicate significant differences at *p* < 0.05 level.

Next, we analyzed the resistance of the *SlRboh*-silenced plants to *B. cinerea* using a detached leaf inoculation assay. The lesions on leaves from TRV2-*SlRbohA*-, TRV2-*SlRbohC*-, TRV2-*SlRbohD*-, TRV2-*SlRbohE*-, TRV2-*SlRbohF*-, TRV2-*SlRbohG*-, or TRV2-*SlRbohH*-infiltrated plants were comparable to those from TRV2-*GUS*-infiltrated plants and wild type (WT) plants (**Figures 4A,B**), whereas the lesions on leaves from TRV2-*SlRbohB*-infiltrated plants were significantly larger at 3 days post inoculation (dpi; **Figure 4A**), showing an approximately 40% larger in size than those in TRV2-*GUS*-infiltrated plants or WT plants (**Figure 4B**). To confirm the disease phenotype observed in TRV2-*SlRbohB*-infiltrated plants, the *in planta* growth of *B. cinerea* was measured by analysis of the transcript level of the actin gene *BcActin* as an indicator of the rate of fungal growth and compared between the TRV-SlRbohB- and TRV-*GUS*-infiltrated plants in whole plant inoculation assays. As shown in **Figure 4C**, the *in planta* growth of *B. cinerea* in TRV2-*SlRbohB*-infiltrated plants increased markedly at 2 and 3 dpi, leading to three times greater than that observed in TRV2-*GUS*-infiltrated plants at 3 dpi. Together, these data demonstrate that silencing of *SlRbohB* attenuated the resistance to *B. cinerea* and *SlRbohB* is therefore required for resistance against *B. cinerea* whereas *SlRbohA*, *SlRbohC*, *SlRbohD*, *SlRbohE*, *SlRbohF*, *SlRbohG*, and *SlRbohH* may not be involved in resistance to *B. cinerea*.

### Transient Expression of *SlRbohB* in *N. benthamiana* Conferred Increased Resistance to *B. cinerea*

**FIGURE 5:**
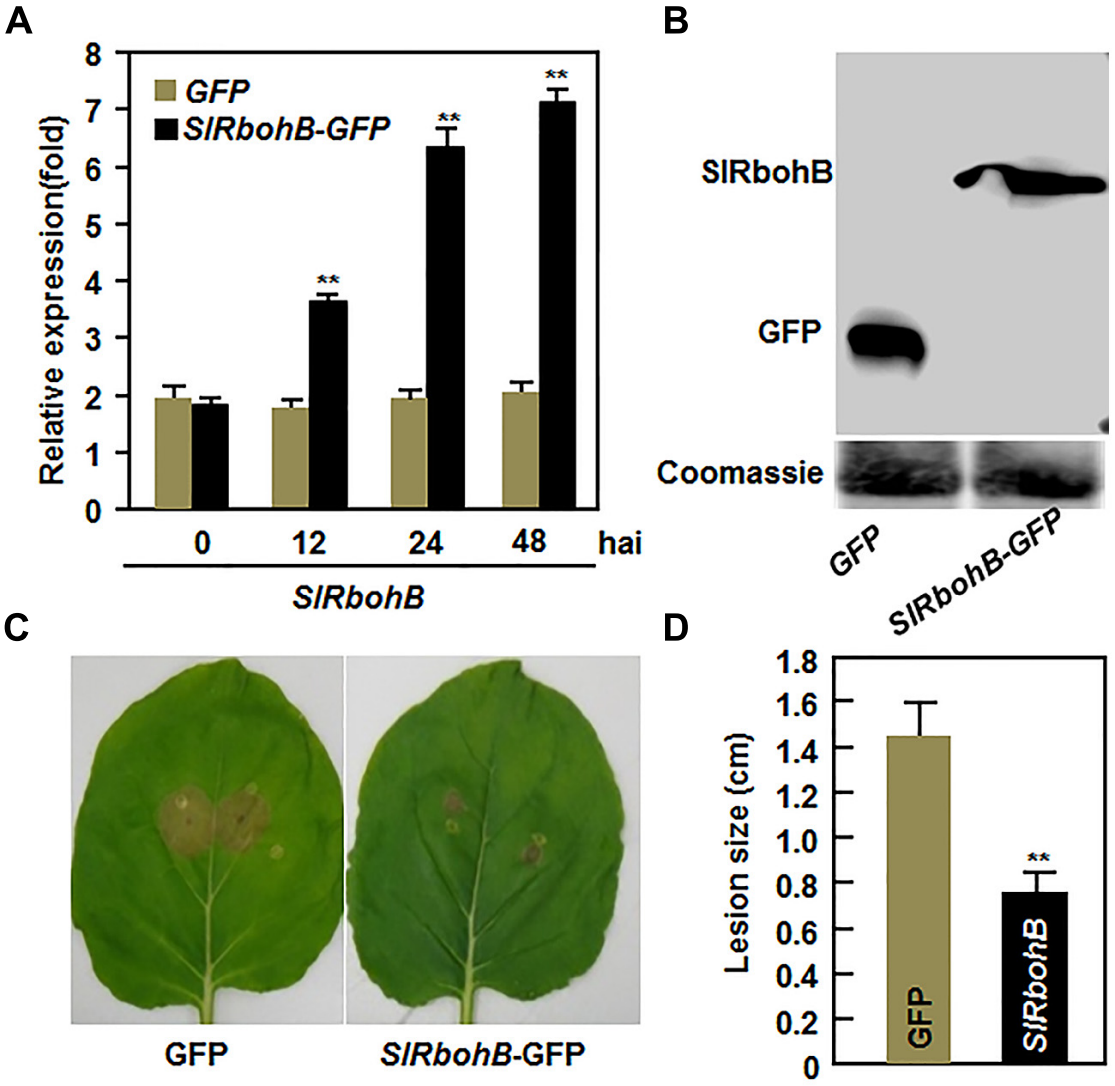
**Transient expression of *SlRbohB* in *Nicotiana benthamiana* conferred an increased resistance to *B. cinerea*.** Agrobacteria harboring pFGC-*SlRbohB* or pFGC-eGFP were infiltrated into leaves of 4-week-old *N. benthamiana* plants and leaf samples were collected for different assays. **(A)** Expression of *SlRbohB* in pFGC-*SlRbohB-*infiltrated leaves. **(B)** The *SlRbohB*-GFP fusion protein in pFGC-*SlRbohB*-infiltrated leaves. Leaf samples were harvested 48 h after infiltration, total soluble proteins were separated by SDS–PAGE, and analyzed by immunoblotting using a GFP-specific antibody. Equal loading of total proteins was verified by Coomassie blue staining. **(C)** Disease symptom on representative leaves. **(D)** Lesion sizes. The pFGC-*SlRbohB*- and pFGC-eGFP-infiltrated leaves were detached at 48 h after infiltration and inoculated by dropping spore suspension (2 × 10^5^ spores/mL). Photos were taken and lesion sizes were measured on a minimum of 10 leaves at 5 days after inoculation. Data presented in **(A,D)** are the mean ± SD from three independent experiments and ** above the columns indicate significant difference at *p* < 0.05 level.

To further confirm the function of *SlRbohB* in resistance to *B. cinerea*, we examined whether overexpression of *SlRbohB* could confer increased resistance to *B. cinerea*. When transiently expressed in *N. benthamiana* leaves, high levels of *SlRbohB* expression and the SlRbohB-GFP fusion protein were detected (**Figures 5A,B**). In disease assays, the lesions on leaves of SlRbohB-infiltrated *N. benthamiana* plants were significantly smaller than those on eGFP vector-infiltrated control plants (**Figure 5C**), leading to approximately 40% of reduction in lesion size, at 5 dpi (**Figure 5D**). These data demonstrate that transient expression of *SlRbohB* in *N. benthamiana* conferred an increased resistance to *B. cinerea*. Therefore, SlRbohB positively regulates the defense response against *B. cinerea*.

### Silencing of *SlRbohB* Attenuated Defense Response to *B. cinerea*

**FIGURE 6:**
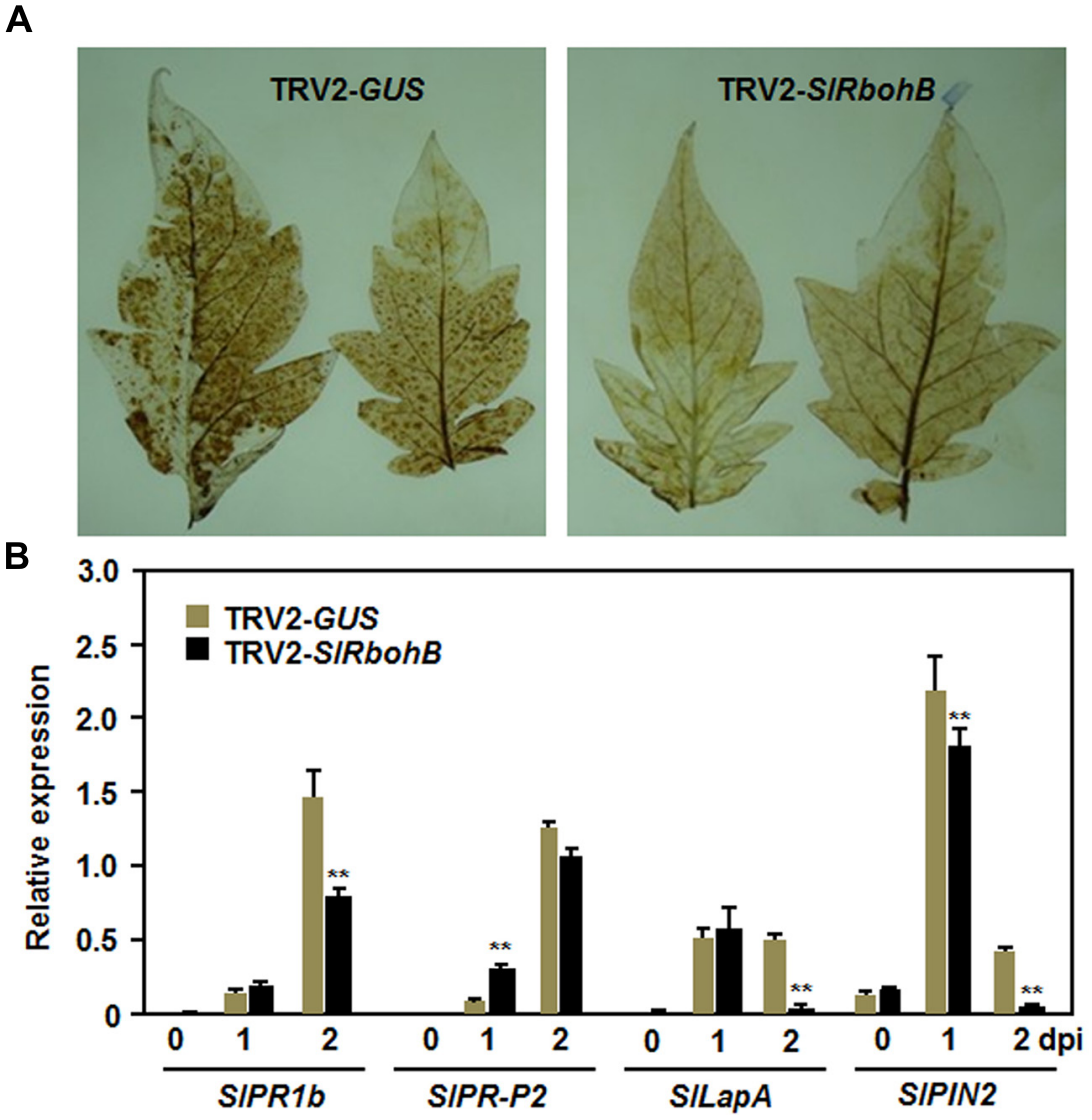
**Silencing of *SlRbohB* attenuated *B. cinerea*-induced defense response.** Two-week-old plants were infiltrated with agrobacteria harboring TRV2-*SlRbohB* or TRV2-*GUS* and the leaves were inoculated by dropping spore suspension (1 × 10^5^ spores/mL) at 4 weeks after agroinfiltration. **(A)** Accumulation of H_2_O_2_ and **(B)** expression of the selected defense genes. Accumulation of H_2_O_2_ was detected by DAB staining at 24 h after inoculation. Expression of the defense genes was analyzed by qRT-PCR and relative expression was shown as folds of the transcript levels at different time points vs. the corresponding values at 0 h after inoculation. Data presented in **(B)** are the means ± SD from three independent experiments and ** above the columns indicate significant difference at *p* < 0.05 level.

To explore the possible mechanism involved in the reduced resistance observed in *SlRbohB*-silenced plants, we analyzed and compared the accumulation of H_2_O_2_ and the expression of defense genes in TRV2-*SlRbohB*- and TRV2-*GUS*-infiltrated plants. No difference in the accumulation of H_2_O_2_, as detected by DAB staining, was detected in leaves of TRV2-*SlRbohB*- and TRV2-*GUS*-infiltrated plants without *B. cinerea* infection (data not shown). A significant accumulation of H_2_O_2_, shown as brown precipitates in leaves, was detected in TRV2-*SlRbohB*- and TRV2-*GUS*-infiltrated plants after infection with *B. cinerea* (**Figure 6A**). However, the accumulation of H_2_O_2_ in the leaves of TRV2-*SlRbohB*-infiltrated plants was reduced compared with that of TRV2-*GUS*-infiltrated plants (**Figure 6A**). Conversely, the expression levels of selected defense genes increased significantly in TRV2-*SlRbohB*- and TRV2-*GUS*-infiltrated plants after infection with *B. cinerea*. However, the kinetic of expression exhibited distinct patterns (**Figure 6B**). After infection with *B. cinerea*, the expression levels of *SlPRP1b*, *SlLapA*, and *SlPIN2* in TRV2-*SlRbohB*-infiltrated plants were comparable to those in TRV2-*GUS*-infiltrated plants at 1 dpi but were significantly decreased at 2 dpi, by reduction of 50% for *SlPR1b* and >90% for *SlLapA* and *SlPIN2* (**Figure 6B**). However, the expression level of *SlPR-P2* in TRV2-*SlRbohB*-infiltrated plants was higher than that in TRV2-*GUS*-infiltrated plants at 1 dpi but the expression levels were similar at 2 dpi (**Figure 6B**). These results indicate that silencing of *SlRbohB* compromised the accumulation of H_2_O_2_ and attenuated the defense response by downregulating the expression of defense genes in tomato upon infection with *B. cinerea*.

### Silencing of *SlRbohB* Attenuated flg22-Induced PAMP-Triggered Immunity (PTI)

**FIGURE 7:**
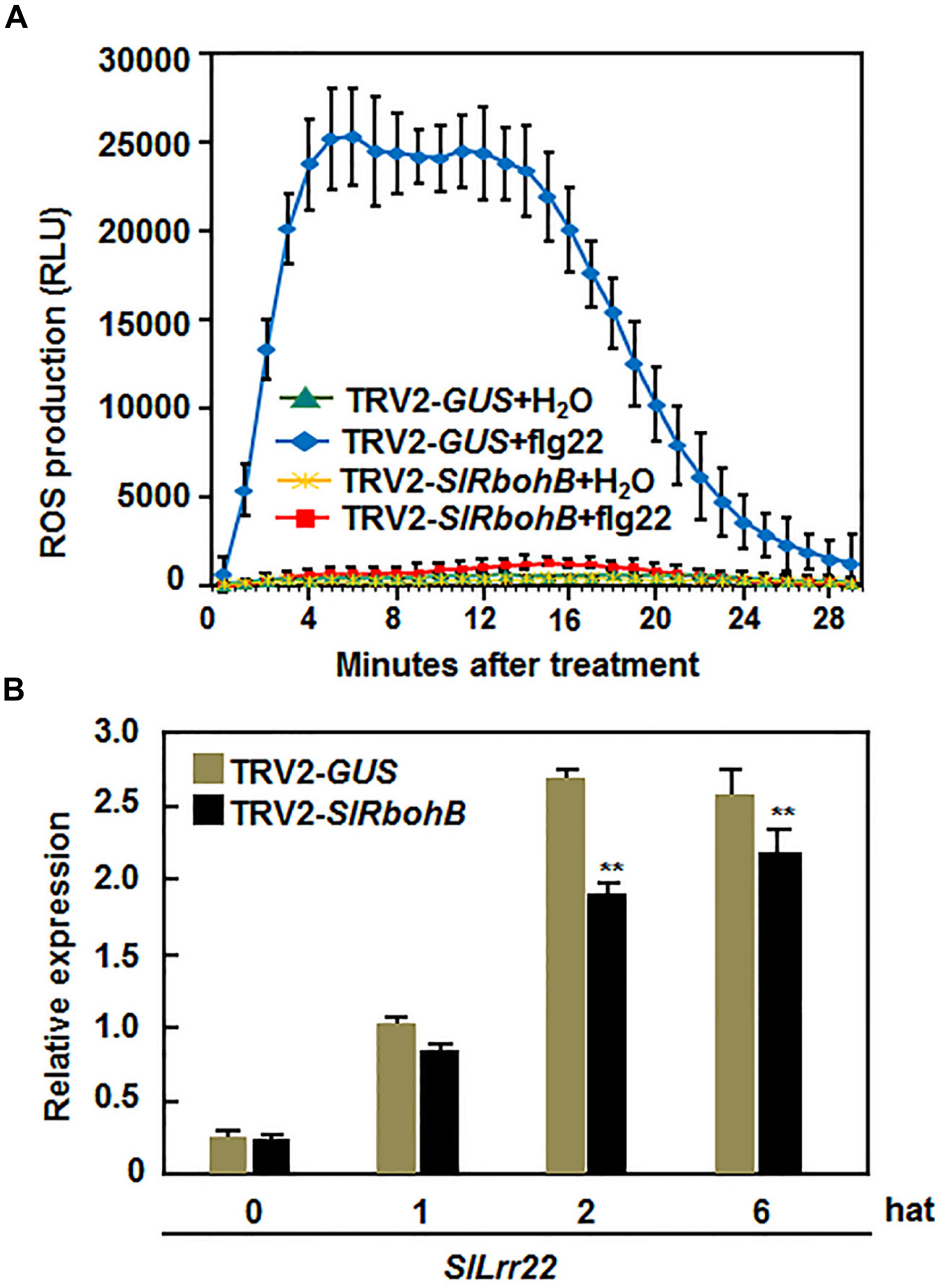
**Silencing of *SlRbohB* attenuated flg22-induced PTI.** Two-week-old plants were infiltrated with agrobacteria harboring TRV2-*SlRbohB* or TRV2-*GUS* and the leaves were collected at 4 weeks after agroinfiltration. **(A)** Detection of flg22-induced reactive oxygen species (ROS) burst. ROS burst was monitored by a luminol-based assay immediately after addition of flg22 (400 nM) or solution. Repeated experiments showed similar results. **(B)** Expression of PTI marker gene *SlLrr22* induced by flg22. Expression of *SlLrr22* was analyzed by qRT-PCR and relative expression was shown as folds of the transcript levels at different time points vs. the value at 0 h after treatment (hat). Data presented are the means ± SD from three independent experiments and ** above the columns indicate significant difference at *p* < 0.05 level.

Several *Arabidopsis* AtRbohs have been shown to play important roles in PAMP-triggered immunity (PTI; Kaur et al., 2014). Thus, we explored whether SlRbohB has a function in PTI by analyzing and comparing the flg22-induced ROS burst and the expression of PTI marker genes in TRV2-*SlRbohB*- and TRV2-*GUS*-infiltrated plants. As shown in **Figure 7A**, a significant ROS burst in the leaves of TRV2-*GUS*-infiltrated plants was detected within 4–18 min, while no such ROS burst was observed in the leaves of TRV2-*SlRbohB*-infiltrated plants after the addition of 400 nM flg22. No ROS burst was observed in the untreated leaves of TRV2-*GUS*- and TRV2-*SlRbohB*-infiltrated plants (**Figure 7A**). The expression level of *SlLrr22*, a PTI marker gene in tomato (Taylor et al., 2012), in TRV2-*SlRbohB*-infiltrated plants was significantly lower than that in TRV2-*GUS*-infiltrated plants at 2 and 6 hours after treatment (hat) with flg22 (**Figure 7B**). These data indicate that silencing of *SlRbohB* also attenuated the flg22-induced PTI, demonstrating a role for *SlRbohB* in PTI.

### SlRbohB is Required for Drought Stress Tolerance

**FIGURE 8:**
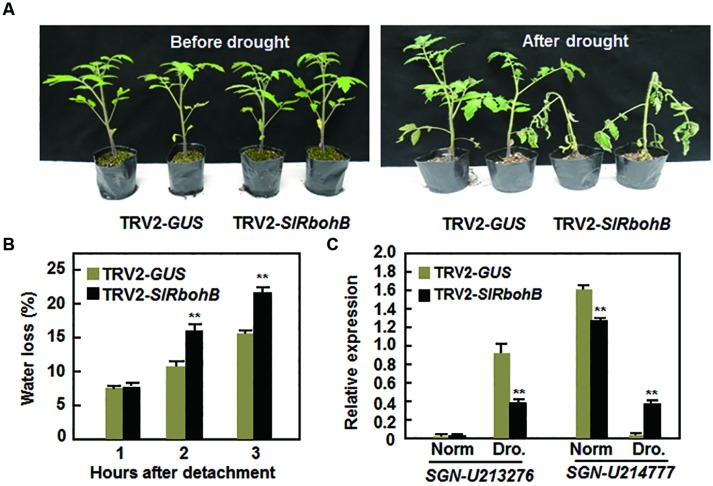
**Silencing of *SlRbohB* led to reduced drought tolerance.** Two-week-old plants were infiltrated with agrobacteria harboring TRV2-*SlRbohB* or TRV2-*GUS* and the TRV2-*SlRbohB*- and TRV2-*GUS*-infiltrated plants were subjected to drought assays at 4 weeks after agroinfiltration. **(A)** Growth performance of the TRV2-*SlRbohB*- and TRV2-*GUS*-infiltrated plants under normal watering conditions (left) and at 10 days after drought stress treatment by withholding watering (right). **(B)** Rates of water loss in detached leaves of the TRV2-*SlRbohB*- and TRV2-*GUS*-infiltrated plants. **(C)** Expression of drought-responsive genes. Leaf samples were collected from the TRV2-*SlRbohB*- and TRV2-*GUS*-infiltrated plants under normal (Norm) and drought (Dro.) conditions and relative expression was shown as folds of that of *SlActin*. Data presented in **(B,C)** are the means ± SD from three independent experiments and ** above the columns indicate significant difference at *p* < 0.05 level.

The involvement of Rbohs in abiotic stress has been documented recently (Kaur et al., 2014). We therefore examined whether SlRbohB has a function in the abiotic response by analyzing the effect of silencing of *SlRbohB* on drought stress tolerance. Under normal watering conditions, the TRV2-*SlRbohB*-infiltrated plants grew as well as the TRV2-*GUS*-infiltrated plants (**Figure 8A**). However, the TRV2-*SlRbohB*-infiltrated plants displayed significant wilting symptom and their leaves began to curl after 10 days of drought stress treatment, whereas the TRV2-*GUS*-infiltrated plants did not show any stress symptoms (**Figure 8A**). The rate of water loss in the leaves of the TRV2-*SlRbohB*-infiltrated plants was >45% greater than that in the leaves of the TRV2-*GUS*-infiltrated plants at 2 and 3 h after detachment (**Figure 8B**). This finding indicates that silencing of *SlRbohB* accelerated water loss in leaves. Under normal watering conditions, the expression level of the drought-upregulated stress-responsive gene *SGN-U213276* (Gong et al., 2010) in the TRV2-*SlRbohB*-infiltrated plants was comparable to that in TRV2-*GUS*-infiltrated plants, while the expression level of *SGN-214777*, a drought-downregulated stress-responsive gene (Gong et al., 2010), was lower in the TRV2-*SlRbohB*-infiltrated plants than that in TRV2-*GUS*-infiltrated plants (**Figure 8C**). After drought stress treatment, the expression of *SGN-213276* was induced in both TRV2-*SlRbohB*- and TRV2-*GUS*-infiltrated plants (**Figure 8C**); however, the expression level of this gene in the TRV2-*SlRbohB*-infiltrated plants was lower than that in TRV2-*GUS*-infiltrated plants (**Figure 8C**). In contrast, the expression level of *SGN-214777* in the TRV2-*GUS*-infiltrated plants was lower than that in TRV2-*SlRbohB*-infiltrated plants under normal watering condition but was significantly reduced as compared with those in the TRV2-*GUS*-infiltrated plants after drought stress treatment (**Figure 8C**). These data indicate that silencing of *SlRbohB* resulted in reduced drought tolerance and thus SlRbohB has a positive function in regulating the drought stress response in tomato.

## Discussion

In this study, we identified eight Rboh family members in tomato (**Table 1**). All SlRboh proteins share characteristic structural features in terms of the presence and organization of functional domains with previously identified plant Rboh proteins (**Figure 1**). Two of the *SlRboh* genes, *SlRbohB* (*SlWfi1*), and *SlRbohG* (*SlRboh1*), have been previously identified (Sagi et al., 2004) and shown to play roles in developmental processes, abiotic stress, and wounding responses (Sagi et al., 2004; Zhou et al., 2012, 2014; Xia et al., 2014). In the present study, we carried out a systemic VIGS-based functional analysis of the SlRboh family in stress responses. Our results demonstrate that SlRbohB plays important roles in resistance to *B. cinerea* and flg22-induced PTI and also functions in drought stress tolerance.

The activity of Rboh proteins may be regulated at both transcriptional and post-transcriptional levels. Rboh family members in various plants have been shown to be induced by wounding, pathogens, or different abiotic stress stimuli (Yoshioka et al., 2003; Proels et al., 2010; Suzuki et al., 2011; Chaouch et al., 2012; Ma et al., 2012; Montiel et al., 2012; Zhou et al., 2012; Pastor et al., 2013; Siddique et al., 2014; Xu et al., 2014). In the present study, we found that some of the tomato *SlRboh* genes are responsive to *B. cinerea* and *Pst* DC3000 (**Figure 2**). Among these pathogen-inducible *SlRbohs*, expression of *SlRbohA*, *SlRbohB*, and *SlRbohC* was induced by both of *B. cinerea* and *Pst* DC3000; whereas expression of *SlRbohE*, *SlRbohF*, and *SlRbohG* was induced only by *B. cinerea* but not by *Pst* DC3000 (**Figure 2**). The differential responsiveness of the *SlRboh* genes to pathogens implies that they play different roles in the response to biotic stress. The significance of the transcriptional regulation of *Rbohs* is supported by several observations that overexpression or silencing of *Rboh* genes resulted in altered resistance to a range of pathogens (Torres et al., 2002, 2005; Perchepied et al., 2010). Although expression of *SlRbohA*, *SlRbohC*, *SlRbohE*, *SlRbohF*, and *SlRbohG* was induced by *B. cinerea* (**Figure 2A**), our data indicate that these SlRbohs may not be involved in resistance to *B. cinerea* as silencing of each of these genes did not affect the resistance to *B. cinerea* (**Figure 3**). Alternatively, it is possible that post-translational modification is required for the enzyme activity of these SlRbohs after the expression is upregulated at the transcriptional level by pathogens or other stimuli. It was previously reported that post-translational regulation of Rboh activity is required for ROS production as overexpression of the *Rboh* gene did not result in constitutive ROS production (Torres et al., 2005; Kobayashi et al., 2012; Asai et al., 2013). Thus, further biochemical experiments are necessary to examine the possibility that post-translational modification is involved in regulating the activity of these SlRbohs, which are required to modulate the generation of ROS in tomato plants upon infection of *B. cinerea*.

It was suggested that Rboh-dependent ROS may contribute to help the necrotrophic pathogens establish colonization in plant tissues (Kobayashi et al., 2012). In our VIGS-based functional analyses, we found that silencing of *SlRbohB* resulted in reduced resistance against *B. cinerea*, as the *SlRbohB*-silenced plants exhibited severer severity of the disease and supported much *in planta* growth of *B. cinerea* as compared with the non-silenced plants (**Figure 4**). This is in contrast with observations that *NbRbohB*-silenced *N. benthamiana* plants displayed increased resistance to *B. cinerea* (Asai and Yoshioka, 2009) and knockout of *AtRbohD*, the *Arabidopsis* homolog of *SlRbohB*, showed increased resistance to *Fusarium oxysporum* (Zhu et al., 2013). However, recent studies found that Rboh-mediated ROS accumulation is not strictly correlated with disease susceptibility to *B. cinerea*. For example, treatment of *Arabidopsis* leaves with oligogalacturonides (OGs) elicited an AtRbohD-dependent ROS burst and subsequently protected plants from attack by *B. cinerea* and the *AtRbohD* mutant plants exhibited an induction of defense genes and an increased resistance to *B. cinerea* after OG treatment (Galletti et al., 2008). Conversely, Rboh-generated ROS are thought to act as one of the earliest signaling events that mediate the activation of immune responses (Kaur et al., 2014). The fact that reduced resistance to *B. cinerea* in the *SlRbohB*-silenced plants (**Figure 4**) is accompanied by a decrease in the *B. cinerea*-induced accumulation of ROS and the expression of defense genes (**Figure 6**) suggest that SlRbohB-dependent generation of ROS should act to activate the defense response rather than promote pathogen infection. This is in agreement with previous observations concerning AtRbohD, which negatively regulates cell death and whose mutant plants reduced the accumulation of ROS induced by *B. cinerea* (Torres et al., 2005). However, whether silencing of *SlRbohB*, closely related to AtRbohD, has an effect on cell death and if so, the relationship between SlRbohB-regulated cell death and the reduced resistance to *B. cinerea* in *SlRbohB*-silenced plants are open questions to be further investigated. Furthermore, we also found that the transient expression of *SlRbohB* in *N. benthamiana* conferred an enhanced resistance to *B. cinerea* (**Figure 5**). Taken together, these data demonstrate that SlRbohB functions as a positive regulator of the defense response to *B. cinerea* in tomato.

During PTI, the ROS burst is an early response that is believed to play important roles in the activation of the immune response (Zipfel et al., 2004; Nicaise et al., 2009). The involvement of Rbohs and Rboh-generated ROS in plant immune responses including PTI has been well established (Torres, 2010; Pastor et al., 2013). flg22, a well characterized PAMP, is perceived by SlFLS2 in tomato (Robatzek et al., 2007). Treatment with flg22 can not only induce globally the expression of defense genes and accumulation of ROS production but also protect plants from subsequent infection with virulent pathogens (Mueller et al., 2012). In the present study, we found that the flg22-induced ROS burst was greatly suppressed in *SlRbohB*-silenced plants (**Figure 7A**). This is similar to the report that the flg22-induced ROS burst in *Arabidopsis AtRbohD* mutant plants was completely abolished (Zhang et al., 2007; Kadota et al., 2014; Li et al., 2014b). The flg22-induced expression of *SlLrr22*, a PTI marker gene in tomato (Taylor et al., 2012), was attenuated in the *SlRbohB*-silenced plants (**Figure 7B**). These experimental data demonstrate that, in addition to the function in resistance to *B. cinerea*, SlRbohB also play a role in regulating PTI in tomato. Recently, it was found that receptor-like cytoplasmic kinase BIK1, a component of the FLS2 immune receptor complex, directly phosphorylates AtRbohD in a calcium-independent manner to enhance ROS generation (Kadota et al., 2014; Li et al., 2014b). Conversely, mutation of the flg22 receptor AtFLS2 abolished the flg22-induced ROS burst and led to enhanced susceptibility to *B. cinerea* in *Arabidopsis* (Zipfel et al., 2004) and early accumulation of ROS was found to be correlated with resistance to *B. cinerea* in the ABA-deficient mutant *sitens* in tomato (Asselbergh et al., 2007). It is thus possible that the attenuated flg22-induced ROS burst is related to or even responsible for the reduced resistance to *B. cinerea* in *SlRbohB*-silenced plants. However, the mechanism of SlRbohB in PTI and the relationship between SlRbohB-mediated ROS burst and resistance to *B. cinerea* need to be investigated further.

Rbohs-generated ROS has been shown to regulate responses to various abiotic stresses such as wounding, light/radiation and ozone exposure, and salinity (Baxter et al., 2014). In *Arabidopsis*, AtRbohD- and AtRbohF-generated ROS is involved in stomatal closure in guard cells, the cold stress response and systemic acquired acclimation (Kwak et al., 2003; Kawarazaki et al., 2013; Suzuki et al., 2013). In the present study, we found that silencing of *SlRbohB* resulted in reduced drought tolerance (**Figure 8A**), indicating that SlRbohB also functions in drought stress tolerance in tomato. This hypothesis is supported by the observations that the detached leaves from the *SlRbohB*-silenced plants exhibited higher rate of water loss (**Figure 8B**) and altered expression of drought-responsive genes *SGN-U213276* and *SGN-U214777*, which were found to be upregulated or downregulated in drought stress, respectively (Gong et al., 2010), under stress condition (**Figure 8C**). Thus, it is possible that silncing of *SlRbohB* attenuated the drought stress response and thereby reduced drought tolerance in tomato. In addition, Rbohs have been shown to be involved in root system development (Foreman et al., 2003; Kwak et al., 2003; Jiao et al., 2013), which may affect the capacity of plants to take up water from the soil. However, silencing of *SlRbohB* did not affect the root system in SlRbohB-silenced plants (data not shown), indicating a limited role for the root system in SlRbohB-regulated drought tolerance.

## Conclusion

Rbohs mediate the generation of ROS, thereby regulating a diverse range of biological processes in plants. The present study focused on the function of the SlRboh family in biotic and abiotic stress responses in tomato. The results from VIGS- and transient expression-based functional analyses clearly demonstrate that, in addition to the previously reported involvement in development and wounding response (Sagi et al., 2004), SlRbohB positively regulates the resistance to *B. cinerea*, flg22-induced PTI, and drought stress tolerance in tomato. Further work in decoding downstream signaling will help elucidate the molecular and physiological mechanisms by which SlRbohB regulates biotic and abiotic stress responses.

## Author Contributions

XL, HZ, LT, LH, and SL carried out most of the experiments. DL performed bioinformatics analysis. DL, XL, and FS designed the experiments. FS and DL wrote the paper. All authors read and approved the final manuscript.

## Conflict of Interest Statement

The authors declare that the research was conducted in the absence of any commercial or financial relationships that could be construed as a potential conflict of interest.

## Footnotes

1 http://solgenomics.net

2 http://pfam.sanger.ac.uk/

3 http://smart.embl-heidelberg.de/
