# Supplementary material for: Tomato SlRbohB, a member of the NADPH oxidase family, is required for disease resistance against Botrytis cinerea and tolerance to drought stress
Source: Front Plant Sci. 2015 Jun 23;6:463. doi: 10.3389/fpls.2015.00463 (PMC4477072; doi:10.3389/fpls.2015.00463)
Supplement: Supplementary file 1 [file Table_1.DOC]

**Supplementary Table S1.** Primers used in this study for different purposes

| Primers | Sequences (5’-3’) | Size (bp) |
| --- | --- | --- |
| ***Cloning of ORF*** | | |
| SlRbohA-F | ATGGAGATCGAAAACACGACAG | 2598 |
| SlRbohA-R | TTAGAAATTTTCTTTATGAAAT |
| SlRbohB-F | ATGCAAAATTCGGAAAATCATC | 2817 |
| SlRbohB-R | TCAAAAATTTTCTTTATGGAAATC |
| SlRbohC-F | ATGCAGTTAATGTCACTTTTTA | 2601 |
| SlRbohC-R | TCACCGTATTTGTTTTGATTGA |
| SlRbohD-F | ATGCAAAATCCAGAAGATCACC | 2574 |
| SlRbohD-R | TCAAAAGTTTTCTTTATGGAAATC |
| SlRbohE-F | ATGGTGCCCATTGACGATGGAG | 2493 |
| SlRbohE-R | CTAGAAATTTTCTTTGTGGAAA |
| SlRbohF-F | ATGTTACCGATTTTTCTCAATG | 2646 |
| SlRbohF-R | TCAAAAGTACTCTTTGTGGAAC |
| SlRbohG-F | ATGAGGGGTTTACCTGGGCATG | 2892 |
| SlRbohG-R | CTAAAAATGTTCTTTGTGAAAC |
| SlRbohH-F | ATGGGAGAAACCCCTATAG | 2451 |
| SlRbohH-R | CTAGAAGTTCTCTTTGTGG |
| ***VIGS constructs*** | | |
| SlRbohA-VIGS-F | CCG GAATTCGAGAGTAGGATTCAGCGGT | 302 |
| SlRbohA-VIGS-R | CCG GAGCTC TAGAGCACGAGCAGCACC |
| SlRbohB-VIGS-F | TGC TCTAGA AGGGAATGATAGAGCGTCG | 333 |
| SlRbohB-VIGS-R | CCG GAGCTC AGCTCTTGAGACACTTGCC |
| SlRbohC-VIGS-F | TGC TCTAGAGGAGCTGCTCGAATTACAGT | 328 |
| SlRbohC-VIGS-R | CCG GAGCTCCTCTCTCAGCGCTTGATTTA |
| SlRbohD-VIGS-F | TGC TCTAGA CCTCCTACACCACCAAATC | 303 |
| SlRbohD-VIGS-R | CCG GAGCTC CAGAGCCACCATCAGTCCT |
| SlRbohE-VIGS-F | TGC TCTAGA GTGTGACAAGAATGGTGATGG | 385 |
| SlRbohE-VIGS-R | CCG GAGCTC CCAAGTGAAAAGTATCATGTTG |
| SlRbohF-VIGS-F | TGC TCTAGA GTATGTCGGAGGAGCAATG | 393 |
| SlRbohF-VIGS-R | CCG GAGCTC CGTTGAGCCGTTGATTTAC |
| SlRbohG-VIGS-F | TGC TCTAGA ATTGATGGTTCTGATCCTGC | 301 |
| SlRbohG-VIGS-R | CCG GAGCTC AGTCTACACCGTTGCCTGTT |
| SlRbohH-VIGS-F | TGC TCTAGA ATTTGGACCCTCTATAGGT | 376 |
| SlRbohH-VIGS-R | CCG GAGCTC TTTGTTGGCTGAAGCACTC |
| ***Transient expression*** | | |
| SlRbohB-GFP-F | GCTCTAGA ATGCAAAATTCGGAAAATCATC | 2817 |
| SlRbohB-GFP-R | TCCCCCGGG TCAAAAATTTTCTTTATGGAAATC |
| ***qRT-PCR*** | | |
| SlRbohA-RT-F | GAGAGTAGGATTCAGCGGT | 173 |
| SlRbohA-RT-R | GCCTCTTTTCGAGCTTGCT |
| SlRbohB-RT-F | AGGGAATGATAGAGCGTCG | 143 |
| SlRbohB-RT-R | CATCGTCATTGGACTTGGC |
| SlRbohC-RT-F | ATGGAAAGCGGTGAGGAATAAA | 133 |
| SlRbohC-RT-R | AGCATTGAACCGACTCTCAAC |
| SlRbohD-RT-F | CCTCCTACACCACCAAATC | 137 |
| SlRbohD-RT-R | GCCCAGTGCTTCAATCTCT |
| SlRbohE-RT-F | GGGTCCTAACATTGTGGTTGT | 130 |
| SlRbohE-RT-R | AGAGTCTCTCCTGCACCTTTA |
| SlRbohF-RT-F | CCTTATCTGCACGAGAGGAAAT | 128 |
| SlRbohF-RT-R | CAGCACATTTGTGTCAGATTCC |
| SlRbohG-RT-F | TGAGGAAGAGAAGCCCAATAAG | 91 |
| SlRbohG-RT-R | CACAAGACCAGAACCCAAATTC |
| SlRbohH-RT-F | CATGTGTGACAAGAATGGTGATG | 109 |
| SlRbohH-RT-R | TGCTGCATGTTTCTTGAAGTTT |
| SlActin-RT-F | CCAGGTATTGCTGATAGAATGAG | 113 |
| SlActin-RT-R | GAGCCTCCAATCCAGACAC |
| SlPIN2-RT-F | CATCTTCTGGATTGCCCA | 106 |
| SlPIN2-RT-R | ACACACAACTTGATGCCCAC |
| SlLapA-RT-F | GGGACTAATGATGTTTGGAA | 109 |
| SlLapA-RT-R | GTGGCAATTTTATTTAGGCA |
| SlPR1b-RT-F | TTTCCCTTTTGATGTTGCT | 96 |
| SlPR1b-RT-R | TGGAAACAAGAAGATGCAGT |
| SlPR-P2-RT-F | CGATCTAAATTGATTTCATAGTACG | 116 |
| SlPR-P2-RT-R | TCGTGAAGGATATACAAAATACA |
| BcActin-RT-F | CGTCACTACCTTCAACTCCATC | 107 |
| BcActin-RT-R | CGGAGATACCTGGGTACATAGT |
| SlLrr22-RT-F | GGGAAGAAGAGAGTTTCCTTGAG | 108 |
| SlLrr22-RT-R | AGTGCAGTCATGGTGCATATAA |
| SGN213276-RT-F | GTCAAACACTGGAAAGCATGAA | 110 |
| SGN213276-RT-R | AGCTGCTCCACTTGTCTTATC |
| SGN214777-RT-F | GGTGTTTGTGCTGACCTACT | 100 |
| SGN214777-RT-R | CTTCCAAATCAGCCAAACCTTC |
